# Validation of the Need for Competing Inventory

**DOI:** 10.3389/fpsyg.2021.721903

**Published:** 2021-10-01

**Authors:** Jesper B. Bugten, Ricardo G. Lugo, Karl Steptoe

**Affiliations:** ^1^Institute for Psychology, Kristiania University College, Oslo, Norway; ^2^Department of Welfare, Management and Organisation, Østfold University College, Hamar, Norway; ^3^Institute for Sport and Exercise Psychology, Loughborough University, Loughborough, United Kingdom

**Keywords:** flow, competitiveness, self-efficacy, need for competing, grit, anxiety, achievement motivation, emotional assessment

## Abstract

Past studies have been conducted on competitiveness and achievement orientation as two noncooperative explanations for achievement motivation and achievement behavior. But a complimentary representation of a competitive-achievement orientation has yet to be explored. This paper developed and validated the need for competing inventory (NCI), and further investigated its relations with achievement orientation, emotional assessment, self-efficacy, grit, anxiety, and flow. The results from the present study support the theoretical construct of the need for competing, in the hope that it will provide a solid foundation for a competitive-achievement orientation, which is suggested to play a significant role in competitive achievement behavior. It is anticipated that the results from the present study will open a debate for including a competitive-achievement orientation in future research with the aim for a stronger predictor for achievement behavior.

## Introduction

Competition is a comprehensive and complex term with several definitions. Competition can be defined as a negative social interdependence, meaning that the situation is competitive if the progress or success of one party interferes with the progress or likelihood of success of other parties (Deutsch, 1949). Within sport psychology, competition is often described as a form of social evaluation; entailing comparison of individual or team performances against one another or against an objective standard of excellence (Martens, 1976). Previous research has highlighted the importance of achievement motivation for the explanation of achievement behavior (Gill and Deeter, 1988). Gill and Deeter (1988) argued that competitive achievement behavior is one of the most prominent features in sport and exercise activities, consisting of widespread individual differences. Some people dread any type of evaluation while others take on all competitive challenges. Those taking on competitive challenges might want to achieve personal goals or perform better than others, whereas for some fun and enjoyment are the primary motives for engaging in competition.

## Related Concepts

McClelland et al. (1953) describe competence as a basic motivator for human behavior, especially achievement behavior. When individual competence is evaluated, they strive to do well or to avoid doing poorly, such as in school or in sports. Maehr and Nicholls (1980) made the important distinction between competitors demonstrating competence with a focus on either self-referenced mastery (task orientation) or on gaining favorable judgments of others (ego orientation). In the early 1980s achievement-goal theory was proposed as a dichotomous model that distinguished between two different goal constructs originating from task and ego orientation: mastery goals and performance goals (Maehr and Nicholls, 1980; Dweck and Elliott, 1983; Nicholls, 1984). Task oriented mastery goals were defined as the striving to develop competence through task mastery and improvement in relation to one’s personal standards, and ego-oriented performance goals were defined as the striving to demonstrate competence gaining favorable judgments of others. In the mid 1990’s Elliot and Harackiewicz (1996) extended the dichotomous model with the inclusion of an approach-avoidance distinction. A more recent progression has established a goal construct organized into a 2 × 2 achievement goal model, and then further developed to a 3 × 2 model by separating mastery-based goals into task-based and self-based categories (Elliot, 1999; Elliot et al., 2011). The 3 × 2 model defines competence through task, self or other goals that are fully crossed with a valence of competence as approach or avoidant that produces six goals: (1) Task-approach (focusing on attaining task-based competence); doing the activity the way it was designed to be done, for example correct execution of a skill like scoring a lot of goals in football. (2) Task-avoidance (focus on avoiding task-based incompetence); not failing to do the activity the way it was designed to be done, for example avoiding poor skill execution. (3) Self-approach (focused on self-based competence); doing better than before, for example, scoring more goals than I usually do. (4) Self-avoidance (focused on self-based incompetence); not doing worse than before, for example, not to score fewer goals than I usually do. (5) Other-approach (focus on attaining other-based competence); doing better than others, for example score more goals than my teammates and (6) Other-avoidance (focused on avoiding other-based incompetence); not doing worse than others, for example, not score fewer goals than my teammates. Mascret et al. (2015) extended this 3 × 2 achievement goal model, that had previously focused on competence striving in education environments, to the sport domain.

Achievement goals are rooted in the need to experience competence, but there might also be another factor explaining achievement behavior, namely competitiveness. Previous research has faced challenges in determining the conceptualization and operationalization of competitiveness, and achievement motivation may have supplemented the focus for a discussion on the definition of competition. In previous research, Vealey (1986) developed the Competitive Orientation Inventory (COI) using Nicholls (1984) achievement-goal theory to help define and operationalize competitiveness. Accordingly, depending on what goal the athlete possesses, competitiveness can take two different forms: performing well (performance) or winning (outcome; Vealey, 1986). Gill and Deeter (1988) argued that although the sport achievement orientation is multidimensional, the precise dimensions cannot be specified with any confidence. Thus, they developed a sport achievement orientation measure; the sport orientation questionnaire (SOQ), which included varied achievement options using an exploratory factor analysis for explaining the specific factor structure. The SOQ operationalized competitiveness as one of three achievement orientations: competitiveness, goal orientation and win orientation. The SOQ operationalizes and defines competitiveness as the desire to enter and strive for success in sport competition (Gill and Deeter, 1988). Gill and Deeter (1988) stressed that their competitiveness factor represents a basic sport-specific achievement orientation. However, the three-factor structure indicated that sports achievement orientation could be further differentiated. Two major types of outcome in sport achievement situation seemed to reflect an orientation from the two other factors; (factor 2, goal orientation) the desire to reach personal goals, and (factor 3, win orientation) the desire to win in interpersonal competition in sport. Gill and Deeter (1988), as well as Vealey (1986), also offered parallels for the achievement-goal theory where factor 2 (goal orientation) has similarities with the mastery orientation, and factor 3 (win orientation) has similarities with the performance orientation. They further operationalized the competitiveness term as one of three components in a sport-specific achievement orientation.

## Competitiveness

The Achievement Goal Questionnaire (AGQ) and SOQ offer two different explanations for achievement motivation. This paper attempts to provide a clearer distinction and relation between the competitive factor and achievement factor and their contribution to achievement motivation, orientation and behavior. By conceptualizing and validating a need for competing measurement tool, and redefining the concept of competitiveness, it might offer a more suitable model for the motivation/orientation for achievement behavior, namely competitive-achievement orientation.

### Competitive-Achievement Orientation

A competitive-achievement orientation is posited to offer a clearer distinction between the need/desire to compete (competitive) and the need/desire for competence (achievement). This is believed to be important because of the proposed differences between the satisfaction of competing and the satisfaction of achieving; wanting to succeed, for example, is qualitatively different from wanting to work to succeed. Establishing a competitive-achievement orientation might help understand these differences as well as the relationship between the competitive-achievement orientations, how these orientations complement each other, and the distinction between individuals with high ambitions for succeeding in competition, and those competitors who are willing to make sacrifices and work to realize their aspirations. Being willing to compete for success, means wanting to compete despite the costs of effort, risk of losing and potential experience of failure. This balance between the competitive orientation (enjoyment of the competition) and achievement orientation (the importance of achieving), might be two closely related interactive dimensions of competitiveness. Thus, being able to generate competitive-achievement motivation and utilizing it for competition could lead to a redefinition of competitiveness.

### The Importance of the Competitive-Achievement Orientation

The purpose for challenging and reconceptualizing a more appropriate and relevant model for explaining achievement orientation and behavior is to improve the measurement and predictability for achievement behavior.

## The Need for Competing and Related Concepts

The need for competing is the competitive spirit or strive in the engagement of competition, and can be defined as the desire to enter, participate and enjoy the competition itself. Evidence suggests, however, that the competitive need has a comprehensive relationship with engagement and aggression, motivation, competitive flow and performance levels. Being competitive means getting more activated/aroused (emotional reactivity prone to aggression) when faced with a competitive task (Karrass et al., 2006; Carré et al., 2009; Carré and Archer, 2018; Wu et al., 2018), which can lead to negative emotions and aggressive behavior (Couppis and Kennedy, 2008; Carré and Olmstead, 2015; Carré and Archer, 2018), but it can also lead to positive emotions, engagement, flow and better performance (Elias, 1981; Csikszentmihalyi and Larson, 1987; Csikszentmihalyi and LeFevre, 1989; Roberts et al., 2007a; Jordet and Hartmen, 2008), depending on variables including enjoyment or frustration (Carré and McCormick, 2008). Moreover, Bossuyt et al. (2014) found that dominant/aggressive behavior was related to approach motivation (approaching a desired stimulus), which is associated with low anxiety levels and high performances, whereas being submissive/non-aggressive was related to avoidance motivation (avoidance of undesired stimulus), which is linked with high anxiety levels and lower performances (Schüler, 2007; Roberts et al., 2007b; Jordet and Hartmen, 2008). Being highly competitive (more engaged and aggressive), therefore, should similarly be linked to reduced anxiety levels, increased flow and better performance levels. Whether or not the competitive task is enjoyable, or frustrating is also expected to be heavily influenced by the competitors’ self-efficacy, the belief in one’s abilities, and are most likely positively related with the need for competing (Bandura, 1977). To be able to compete the individual must have some belief in their ability to make an impact in the competitive task, otherwise it is not competing, just losing.

Research to date has predominantly focused on the association between achievement behavior and grit [Grit Scale (GS) – Duckworth et al., 2007], self-efficacy (General Self-efficacy Scale – Scholz et al., 2002), flow (Short Dipositional Flow Scale – Jackson et al., 2008; Martin and Jackson, 2008), anxiety (Sport Anxiety Scale – Smith et al., 2006), emotional assessment [Self-Assessment Manikin (SAM) – Bradley and Lang, 1994], and achievement orientation (Achievement Goal Questionnaire – Mascret et al., 2015), but the construct validity and relation of the need for competing has yet to be explored.

## Objective

Due to the lack of research on the trait concept of the need for competing and its relation to grit, self-efficacy, flow, anxiety, emotional assessment, and achievement orientation, the present study aimed to explore this phenomenon and its potential associations. Based on the cited theories and research, the hypothesis that the need for competing is a valid construct and correlates with grit, self-efficacy, flow, anxiety, emotional assessment, and achievement orientation, was tested. In study 1, the first prediction was that the items in the need for competing inventory (NCI) would all measure the same factor, suggesting that they all measure the construct of the need for competing. In study 2, for further validation of the NCI, the second prediction was that the need for competing would be negatively related to anxiety, and positively related with positive valanced (approach-motivated) self, task and others goal (achievement orientation), emotional assessment, self-efficacy, grit, and flow. The third predication was that the elite prospect students were expected to score higher on the NCI than the students specializing in sports and the general high school education students. Validating the concept of the need for competing by validating the NCI and finding its suggested correlations, might offer a competitive orientation related to the achievement orientation. It is hoped that the suggested representation of the competitive-achievement orientation will give a more distinct and clearer explanation for motivation and orientation for achievement behavior.

## Method

### Study 1

#### Methods

In study 1 the aim was to develop the items for and examine the factor structure and internal consistency of the NCI.

#### Sample Description

In order to minimize demands for the sample size necessary to achieve statistically significant results in study 1, *a priori* power analysis was conducted, using G^∗^power (Faul et al., 2009), which required a sample size of *N* = 84 to achieve a.3 correlational effect size. The NCI was tested on a total of 109 voluntarily participants consisting of 1^*st*^ and 2^*nd*^ year psychology students, undergraduates at the Inland Norway University of Applied Sciences. The testing was conducted in classroom settings and the participants were divided in two separate classes. Nine of the participants withdrew leaving a total of 100 participants who completed the study.

#### Procedure and Measure Development

The NCI was designed by following the guidance of Devellis (2003) for scale development. The need for competing was determined to be measured (step 1) before seven relevant items were generated (step 2) into the NCI self-report questionnaire (step 3); Q1, *“I always seek challenges, not for the outcome, but for the competition itself”*; Q2, *“I’ll rather seek unachievable challenges than no challenges”*; Q3, *“I’m always willing to compete, despite it being a big chance of losing”*; Q4, *“I always choose to work hard to be able to compete, rather than avoid the extra work and not be able to compete”*; Q5, *“I will stop competing when the goal is achieved”*; Q6, *“Competing is always more satisfying than the potential outcome of a competition”*; Q7, *“I always have a greater need for competing, rather than ensuring the avoidance of failure”*.

The initial item pool was reviewed by research colleagues (step 4), where the inclusion of validated items was considered (step 5) before administering the items to a development sample (step 6). Participants were informed that they would be shown statements that represented types of beliefs that they may have of themselves, and they were instructed to respond on a Likert scale from 1 (strongly disagree) to 5 (strongly agree), where higher scores indicate higher need for competing. For increased test validity a reversed score on question 5 was included (Q5) with the aim if reducing chances of acquiescence bias and boredom (Couch and Keniston, 1960; Baumgartner and Steenkamp, 2001; Podsakoff et al., 2003). After the completion of the tests, the items were evaluated (step 7) and the scale length were optimized (step 8) whereas five of the items were chosen to represent the need for competing questionnaire.

#### Ethical Considerations

The participants gave informed consent to participate and were informed that they had the right to withdraw their information at any time during the survey. The Norwegian Centre for Research Data (NSD) evaluated that the study did not need to seek any further ethical approval, considering that the data was anonymous.

#### Results

The seven items were administered to 109 participants (psychology students), with a 100 participants completing, and the resultant data were factor analyzed. SPSS v.25 was used to perform exploratory factor analysis (EFA) to help determine what the items underlying structure was. The reliability statistics reveals a high internal consistency (Cronbach’s *α* = 0.769) within all the seven items on the questionnaire (Table 1).

**TABLE 1:** Item-total statistics of the NCI.

	**Scale mean if item deleted**	**Scale variance if item deleted**	**Corrected item-total correlation**	**Squared multiple correlation**	**Cronbach’s alpha if item deleted**
Q1	18.33	16.951	0.668	0.483	0.706
Q2	18.57	17.500	0.496	0.285	0.740
Q3	18.19	18.701	0.396	0.235	0.759
Q4	18.02	18.181	0.493	0.300	0.740
Q5	18.22	17.891	0.409	0.228	0.760
Q6	18.59	19.517	0.396	0.192	0.758
Q7	18.60	16.323	0.595	0.405	0.717

The Principal Component Analysis was the factor extraction method used to form uncorrelated linear combinations of the observed variables. The successive components explain progressively smaller portions of the variance and are all uncorrelated with each other. This method is used to obtain the initial factor solution (Osborne et al., 2008). Component 1 with a total score of 2.996, component 2 with a total of 1.016 and cumulative percent of 57.323, which is higher than 1 eigenvalue, and the remaining components under 1 eigenvalue.

An oblique promax rotation was performed and the NCI items were shown to have 2 distinct factors. Further on we retained all the factors whose eigenvalues were lower than 1, and therefore removed Q3 *“I’m always willing to compete, despite it being a big chance of losing”* and Q6 *“Competing is always more satisfying than the potential outcome of a competition”* to support the hypothesized one factor structure (Table 2). Overall, this left us with a total of 5 items on the NCI, still with a high consistency (Cronbach’s *α* = 0.759).

**TABLE 2:** Component Loadings of the NCI.

	**RC1**	**RC2**	**Uniqueness**
Q1	0.765		0.323
Q2	0.446		0.561
Q3		0.873	0.306
Q4	0.715		0.505
Q5	0.855		0.422
Q6		0.753	0.443
Q7	0.661		0.427

*Applied rotation method is promax.*

### Study 2

#### Methods

In study 2 the aim was to further validate the NCI by examining the relationships between the need for competing factor and other key variables in the motivation literature, namely achievement orientation, emotional assessment, self-efficacy, grit, anxiety, and flow. As mentioned, these variables are thought to be linked because of the theoretical underpinning of the need for competing, and as a result of previous research that has highlighted their association (Elias, 1981; Karrass et al., 2006; Roberts et al., 2007a; Carré and McCormick, 2008; Couppis and Kennedy, 2008; Jordet and Hartmen, 2008; Carré et al., 2009; Bossuyt et al., 2014; Carré and Olmstead, 2015; Carré and Archer, 2018; Wu et al., 2018).

#### Sample Description

*A priori* power analysis using G^∗^power was also conducted for study 2, revealing a requirement of a sample size of *N* = 252 to achieve an effect size of.25. Gathered, there were a total of 365 participants in study 2.

If the NCI does assess competitive orientation, then individuals who score higher on the need for competing measure should be more likely to enroll in the competitive classes and participate in competitive sports, rather than individuals who score lower on the need for competing. An individual’s competitive achievement orientation should exert some influence although several other factors might influence the individual competitive achievement orientation also (e.g., abilities, competitive environment).

#### Ethical Considerations

As in study 1, in study 2 the participants gave informed consent to participate and were informed that they had the right to withdraw their information at any time during the survey. The NSD concluded that the study did not require any further ethical approval, considering that the data was anonymous.

#### Procedures and Measures

Testing several undergraduate classes in large group settings, the participants completed a questionnaire containing all the constructs consisting of the NCI, AGQ, GSE, GS, SAM, Sport Anxiety Scale 2 (SAS 2), and Short Dispositional Flow Scale (SDFS).

#### The Need for Competing Inventory

The NCI developed in study 1, consisting of five items and a Likert scale from 1 to 5. The NCI was also used to measure participants need for competing level in study 2, where the items had an acceptable internal consistency (*α* = 0.681).

#### Achievement Goal Questionnaire

The AGQ (Mascret et al., 2015), was translated into Norwegian and adjusted to the sport specific domain, consisting of three items for each of the six goals, resulting in a total of 18 items. The translated sport specific AGQ was further tested on 43 participants (third year BSc psychology students) in a classroom setting. The data were then analyzed with a reliability test showing a high internal consistency in achievement goals: task-approach (*α* = 0.689), task-avoidance (*α* = 0.842), self-approach (*α* = 0.675), self-avoidance (*α* = 0.765), other-approach (*α* = 0.953), and other-avoidance (*α* = 0.948) goals. After the translation the AGQ was assessed in study 2. The scores were gathered still with a high overall internal consistency (*α* = 0.940).

#### General Self-Efficacy Scale

The original general self-efficacy scale was developed by Jerusalem and Schwarzer (1979), and later translated into 33 languages (Schwarzer and Jerusalem, 2010). The GSE has shown high validity in numerous domains and across cultures (Luszczynska et al., 2005). The Norwegian translated GSE scale was assessed, consisting of ten likert-scale items from 1 to 4, where higher scores indicate higher self-efficacy (Scholz et al., 2002). According to Scholz et al. (2002), there is support for an internal consistency (*α* = 0.75 −0.91) revealed in several studies. In the present study the internal consistency was also acceptable (*α* = 0.845).

#### Grit Scale

A Norwegian translation of Duckworth et al.’s (2007) Grit scale was also conducted in study 2, with an acceptable internal consistency (*α* = 0.769). Consisting of twelve items, and a likert scale from 1 to 5. Question 1, 2, 5, 7, 10, and 11 was reversed, as the original, for reducing chances of acquiescence bias and boredom (Couch and Keniston, 1960; Baumgartner and Steenkamp, 2001; Podsakoff et al., 2003).

#### The Self-Assessment Manikin

The Self-Assessment Manikin is a picture-oriented questionnaire developed to measure emotional responses like valence/pleasure of the response (from positive to negative), perceived arousal (from low to high levels), and perceptions of dominance/control (from low to high levels) (Bradley and Lang, 1994). The three items in SAM were translated into Norwegian and used in the study 2 without pictures in the items, showing an acceptable internal consistency (*α* = 0.666). Similarly, as the original, it was used a 9-point scale measuring each item.

#### The Sport Anxiety Scale 2

A Norwegian translated version of the SAS 2, originally from Smith et al. (2006), was assessed for study 2. Consisting of 15 items measuring three dimensions (somatic, worry, and concentration) of the athletes experienced anxiety relating to one’s sport. The somatic dimension refers to the bodily experienced anxiety symptoms, while the cognitive dimensions refers to the psychological experienced anxiety symptoms of worrying and concentration disruption before or while the participant competes (Smith et al., 2006). After assessing the questionnaire in study 2 the internal consistency was acceptable (*α* = 0.882). The anxiety dimension also came out internally consistent: somatic (*α* = 0.828), worry (*α* = 0.862), and (*α* = 0.779).

#### The Short Dispositional Flow Scale

The Short Dispositional Flow Scale (Jackson et al., 2008; Martin and Jackson, 2008), was translated in Norwegian and tested on 43 participants (third year BSc psychology students) in a classroom setting, showing a satisfactory internal consistency (*α* = 0.704). The questionnaire consists of 9 items measuring each 9 dimensions of the participants flow experiences related to his/her activity.

#### Results

The need for competing were correlated with the flow, grit, self-efficacy, emotional assessment, achievement goals, and anxiety variables. 365 participants participated in study 2 (Table 3) with a total of 208 elite prospect students scoring highest on the need for competing (M = 17.91, SD = 3.84), 77 students specializing in sports scoring second highest on the need for competing (M = 16.49, SD = 4.02), and 80 general high school education students scoring lowest on the need for competing (M = 14.85, SD = 5.4). With only 347 completing the sport anxiety scale 2, the remaining tests were completed by all the 365 participants.

**TABLE 3:** Descriptives – NCI and educational program.

**Educational program**	**Mean**	**SD**	**N**
Elite sports	17.91	3.844	208
General sports	16.49	4.025	77
Regular school program	14.85	5.401	80

#### Converging Evidence

Using a correlation matrix (Table 4), the need for competing was found to be significant positively related to the achievement orientation (*r* = 0.388, *p* < 0.001), self-efficacy (*r* = 0.561, *p* < 0.001), flow (*r* = 0.691, *p* < 0.001), and emotional assessment: valence (*r* = 0.352, *p* < 0.001), activation (*r* = 0.236, *p* < 0.001), and control (*r* = 0.309, *p* < 0.001).

**TABLE 4:**
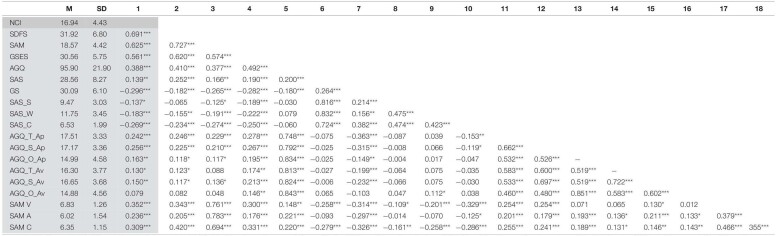
Descriptive statistics and correlations for study variables.

* *p* < 0.05, ** *p* < 0.01, *** *p* < 0.001 Column 1 is highlighted to show the correlation with the NCI and the other factors. NCI, Need for Competing Inventory; GS, Grit Scale; AGQ, Achievement Goal Questionnaire, T(Task), S(Self), O(Other), Ap(Approach), and Av(Avoidance); GSES, General Self-Efficacy Scale; SDFS, Short Dispositional Flow Scale; SAM, Self-Assessment Manikin, V(Valence), A(Arousal), C(Control); SAS 2, Sport Anxiety Scale, S(Somatic), W(Worry), and C(Concentration).

#### Divergent Evidence

Table 4 also revealed that the need for competing was significant negatively related to grit (*r* = −0.296, *p* < 0.001), and anxiety: somatic (*r* = −0.137, *p* < 0.05), worry (*r* = −0.183, *p* < 0.001), and concentration (*r* = −0.269, *p* < 0.001).

The NCI correlated with the different goal dimensions in achievement orientation. The data results revealed a significant positive relation between need for competing and the task approach goal (*r* = 0.242, *p* < 0.001), the self approach goal (*r* = 0.256, *p* < 0.001), and other approach goals (*r* = 0.163, *p* < 0.01), the task avoidance goal (*r* = 0.130, *p* < 0.05) and self avoidance goal (*r* = 0.150, *p* < 0.01). The positive valanced (approach) goals had the strongest positive relations, while the negative valanced (avoidance) goals had a weaker positive correlation to the need for competing. However, the other avoidance goal had the lowest relation and turned out to be non-significant (*r* = 0.079, *p* > 0.05).

**FIGURE 1:**
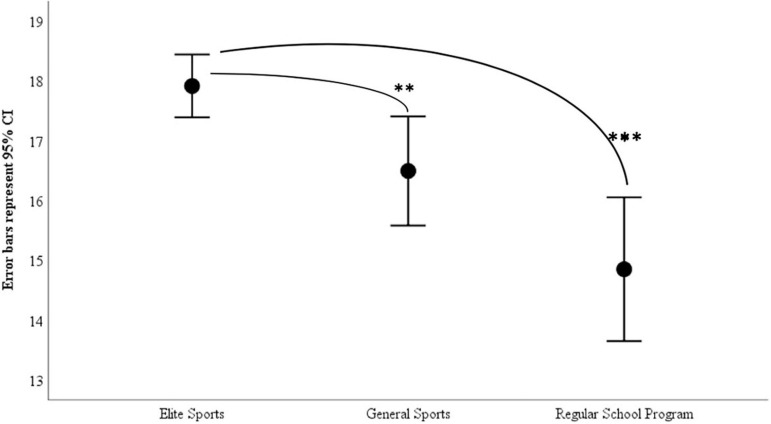
NCI Scores for Different Educational Programs. ^∗^ *p* < 0.05, ^∗∗^ *p* < 0.01, ^∗∗∗^ *p* < 0.001. The figure demonstrating that elite prospect students score greater than the students in general sports and regular school program.

A one-way ANOVA revealed there was a significant difference in the amount of the need for competing score at the *p* < 0.001 level for the three groups with a medium effect size [*F*(2, 362) = 15.42, *p* < .001, η^2^ = 0.079]. As demonstrated in Tables 5, 6 and Figure 1, the elite prospect students scored significantly higher on the need for competing than the general education students (*MD* = 3.06, *p* < 0.001), as well as a significantly higher score difference than the high school students specializing in sports (*MD* = 1.42, *p* < 0.05). The students specializing in sports scored higher on need for competing than the general education students, although not significantly (*MD* = 1.64, *p* > 0.05). These results seem to support previous assumptions that highly competitive individuals seek highly competitive environments.

**TABLE 5:** Results of the NCI within the respective educational programs.

**Educational program**	**Marginal Mean**	**SE**	**Lower CI**	**Upper CI**	**t**	**p**
Elite prospects	17.91	0.296	17.33	18.50	60.53	< 0.001
Sport students	16.49	0.486	15.54	17.45	33.91	< 0.001
General students	14.85	0.477	13.91	15.79	31.12	< 0.001

**TABLE 6:** *Post hoc* comparisons of the NCI and educational programs.

			**95% CI for Mean Difference**					
		**Mean Difference**	**Lower**	**Upper**	**SE**	**t**	**Cohen’s d**	**p_*scheffe*_**
Elite sports	General sports	1.420	0.080	2.760	0.569	2.494	0.365	0.046
	Regular school program	3.063	1.742	4.385	0.561	5.456	0.707	<0.001
General sports	Regular school program	1.644	0.040	3.247	0.681	2.412	0.344	0.056

*Cohen’s d does not correct for multiple comparisons.*

## General Discussion

### Findings

#### Study Purpose

The purpose of this study was to further explore the need for competing and propose a more accurate representation of the competitive-achievement orientation to give a more distinct and clearer explanation for motivation and orientation for achievement behavior in the future.

#### Study 1 – Scale Development

The first prediction, in study 1, was that the items in the NCI would all measure the same factor, named the need for competing. An exploratory factor analysis revealed that two of the items seemed to be measuring another factor and was later removed, leaving 5 remaining items as valid measures of the need for competing factor.

#### Study 2 – Validation

An acceptable factorial validity and reliability do not ensure that the scale is measuring the construct it is designed to measure (Smith et al., 2006). It is vital to specify relations with other theoretically related and unrelated constructs, by attaching the underlying construct in a nomological network (Cronbach and Meehl, 1955), which implies assessing both convergent and discriminant aspects of construct validity (Campbell and Fiske, 1959), leading us to the second prediction. The second prediction was that the NCI would be negatively related to anxiety, and positively related to positive valanced (approach-motivated) self, task and others goal (achievement orientation), emotional assessment, self-efficacy, grit, and flow.

The NCI and AGQ total score were positively correlated (Table 2), however, the prediction that the need for competing were going to be significant positively related to positive valanced (approach-motivated) self, task and others goal (achievement orientation), were partially supported (see Table 4). The positive valanced achievement goals were in fact more positively related than the negative valanced avoidance goals. Although there was no expectation of a positive relation with the need for competing and avoidance achievement goals, the task avoidance goal and self-avoidance goal had a weaker positive correlation to the need for competing, which is understandable since a highly competitive individual might have higher standards toward oneself and find the competitive standards more essential than the average person. However, the other avoidance goal had the lowest relation and turned out to be non-significant. Although, the prediction gets nuanced, the theoretical explanations remain covering the reason for that a highly competitive individual is more frequent approach motivated (Bossuyt et al., 2014).

The relation between NCI and emotional assessment (SAM) had also a positive relation, confirming the suspicion that being competitive are closely related to his/her perceived emotions and emotional response (Wu et al., 2018). This strong positive relation suggests that being highly competitive might be an indication of scoring higher on positive valence, higher activation of excitement, and the perceived control, the feeling of dominating rather than being dominated in competition (Bradley and Lang, 1994). This supports the theoretical implication and previous research that being highly competitive might be associated with higher testosterone, thus influencing their emotional reactivity levels affecting their engagement and aggressive behavior (Simon and Lu, 2006; Nelson and Trainor, 2007; Carré and McCormick, 2008; Carré et al., 2009; Bossuyt et al., 2014; Carré and Olmstead, 2015; Wu et al., 2018). The emotional assessment and self-efficacy were also significantly positively correlated, suggesting that the belief in one’s abilities indicates a feeling of control and domination. Self-efficacy was also strongly positively related to the NCI, furtherly confirming the assumption that being competitive might indicate a more frequently approach motivation because of the individual’s belief in his/her own ability to handle/cope with the competitive task (Bandura, 1977; Bossuyt et al., 2014). Hence, stressors are as mentioned more frequently perceived as benign appraisals (Lazarus and Folkman, 1984; Lazarus, 1991, 1998, 1999). Also, as a result of being competitive, the competitive tasks are more likely to be perceived as meaningful, increasing the tendency of benign appraisal, which again is important for being engaged. Being engaged and believing in one’s abilities as resources to cope with the competitive task, are important elements for reaching a flow state. A flow state is more likely to happen when the challenge and skillset is well balanced, but when unbalanced it is more likely cause frustration and/or anxiety, hence aggressive behavior (Csikszentmihalyi, 1975, 1990, 2000; Csikszentmihalyi and Larson, 1987; Csikszentmihalyi and LeFevre, 1989).

Flow had the strongest positive relation with the need for competing, even stronger than achievement orientation (Table 4), confirming the assumption of the importance of the competitive factor (need for competing) in enjoying the participation of competitive tasks and reaching a highly engaged and focused psychological state. Flow had as expected from past research (Csikszentmihalyi, 1975, 1990, 2000; Stavrou et al., 2015), also in this present study significant strong positive correlations with achievement orientation, self-efficacy and emotional assessment, and a negative relation to the three anxiety dimensions, and a somewhat surprisingly negative correlation with grit (see Table 4).

As expected, the prediction of a negative relation between anxiety and the NCI was confirmed. This further strengthens the theoretical and empirical explanations that a highly competitive individual will more frequently respond in an aggressive fight response, rather than in an anxious flight response (Cannon, 1914; Bossuyt et al., 2014; Wu et al., 2018). Grit was negatively correlated with the NCI. This might suggest that being competitive (need for competing) does not necessarily mean that the individual contains a highly persistence for reaching his/her goals in competition (grit), but the combination of the two traits might be even more associated with successful competitive behavior than past research has revealed (Elias, 1981; Duckworth et al., 2007; Roberts et al., 2007a; Jordet and Hartmen, 2008). A possible explanation for the negative relation between grit and NCI might be that a highly competitive individuals will more often find competitive tasks attractive, hence making it harder to stay focused and resistant in only one specific competition, or to only one specific goal. Another plausible explanation might be that although being competitive contributes to positive emotions, engagement, flow and better performances when faced with challenging and reachable competitive tasks (Elias, 1981; Roberts et al., 2007a; Jordet and Hartmen, 2008), it can also contribute to negative emotions and aggressive behavior when faced with unreachable and frustrating competitive tasks (Couppis and Kennedy, 2008; Carré and Olmstead, 2015; Carré and Archer, 2018). The fact that highly competitive individuals turned out to be less persistent in reaching their competitive goals can simply be a result of poor goalsetting skills. Guiding the competitive individual when facing competitive tasks by setting challenging and reachable goals with the possibility to advance in higher levels later might help to keep the competitive goals in the same competition interesting and enjoyable (Burton et al., 2001).

In the final prediction, the elite prospect students were expected to score higher on the NCI than the students specializing in sports and the general high school education students. Study 2 confirmed the prediction, which further supports the assumption that highly competitive individuals are more likely to seek and participate in competitive environments. It also raises the question of the importance of the need for competing for successful achievement behavior.

### Limitations

Although some minor weaknesses have been mentioned in this paper, four major limitations are acknowledged in the current research. First, relying exclusively on a self-report questionnaire to measure the need for competing, which is an instrument with several limitations (Lucas and Baird, 2006). Although confidentiality was assured in both studies, some participants may have been more motivated than others to look good, leaving the NCI vulnerable to social desirability bias. Also, despite the fact that the five items in the NCI have a satisfactory internal validity, developing more items would still strengthen the validity of the questionnaire. The third limitation is that the current findings do not reveal how the need for competing is related to other converging and diverging variables known to predict achievement behavior, such as testosterone (Simon and Lu, 2006; Nelson and Trainor, 2007; Carré and McCormick, 2008; Carré and Olmstead, 2015), optimistic explanatory style (Seligman and Schulman, 1986), emotion regulation (Karrass et al., 2006), and emotion-focused coping and reappraisal (Oakland and Ostell, 1996). Finally, there is concern when using students as samples for psychological studies regarding issues of representativeness, generalizability, and comparability of results, as students are usually more educated than the general public (Henrich et al., 2010; Hanel and Vione, 2016).

### Future Research

Based on the current study, some future directions need to be highlighted. The vast majority of the studies are basing achievement orientation as a main predictor for achievement behavior (McClelland et al., 1953; Maehr and Nicholls, 1980; Dweck and Elliott, 1983; Nicholls, 1984; Elliot, 1999; Elliot et al., 2011; Mascret et al., 2015). The evidence presented in this study argues the case for the necessity of a competitive orientation as well for a better prediction of achievement behavior. Since competitiveness has been mainly associated with the desire to win at all cost, which could lead to bad sportsmanship and cheating, future studies are suggested to further investigate how the need for competing (enjoyment of competition itself rather than outcome) might lead to less anti-social behaviors in sport, and proposedly in other domains.

For future studies it is also suggested a bigger set of items in the NCI for increasing its validation, as well as a further exploration of more converging and diverging relational factors. For further investigation and validation of the need for competing construct it is also suggested that the NCI should be further analyzed with both the exploratory factor analysis and confirmatory factor analysis. Including the confirmatory factor analysis for future research might help finding the factor structure of the need for competing. Finally, future research should involve larger and more varied samples to improve representativeness, generalizability, and comparability.

## Conclusion

Studies have been conducted on competitiveness and the achievement orientation as two noncooperative explanations for achievement motivation and achievement behavior. But a complimentary representation of a competitive-achievement orientation was yet to be explored, and also the objective of the current study. By developing and validating the NCI, and additionally investigate its relations with achievement orientation, emotional assessment, self-efficacy, grit, anxiety, and flow, for further validation, gave a solid foundation for a competitive orientation and its relation to the achievement orientation. This opened a possibility to include the competitive orientation with the achievement orientation. Although, the findings should be carefully interpreted due to limitations in the present data. These results encourage debate for including a competitive-achievement orientation in future research with the aim for a stronger predictor for achievement behavior.

## Data Availability Statement

The original contributions presented in the study are included in the article/supplementary material, further inquiries can be directed to the corresponding author.

## Ethics Statement

The studies involving human participants were reviewed and approved by The Norwegian Centre for Research Data (NSD). The patients/participants provided their written informed consent to participate in this study.

## Author Contributions

RL and KS contributed with guidance surrounding method design, data analysis, and proofreading. All authors contributed to the article and approved the submitted version.

## Conflict of Interest

The authors declare that the research was conducted in the absence of any commercial or financial relationships that could be construed as a potential conflict of interest.

## Publisher’s Note

All claims expressed in this article are solely those of the authors and do not necessarily represent those of their affiliated organizations, or those of the publisher, the editors and the reviewers. Any product that may be evaluated in this article, or claim that may be made by its manufacturer, is not guaranteed or endorsed by the publisher.
